# The Principles and Practice of Modern Surgery

**Published:** 1900-07

**Authors:** 


THE PRINCIPLES AND PRACTICE OF MODERN SURGERY. For the Use of Students and Practitioners of Medicine and Surgery. By JOHN B. ROBERTS, M. D, Professor of Anatomy and Surgery in the Philadelphia Polyclinic; Mütter Lecturer on Surgical Pathology of the College of Physicians of Philadelphia. New (2d) and revised edition. In one very handsome 8vo volume of 838 pages, with 474 engravings and 8 plates in colors and monochrome. Philadelphia: Lea Brothers & Co. 1900. [Cloth, $4 25 net; leather, $5 25 net.]

In the second edition of this work the author has not only revised the text throughout but has rewritten and enlarged several sections, notably those on appendicitis, diseases and injury of the joints and dislocations. The object of the author has been to write an essentially practical work, giving only the generally accepted operative procedures and surgical principles of the present time. Much space is devoted to the consideration of modern pathology and its teachings. Theories, traditional views and operations, new methods of questionable value and references to literature have been entirely omitted. Consequently, the book is compactly and succinctly written. The style throughout is clear and direct and especially suitable for the student.

The chief fault to be found in the work is a want of completeness —a frequent omission of minor but often important details, a fault so common and yet so easily remedied by the addition of a word phrase or short sentence. This would frequently compel the reader to look elsewhere for additional or special points. For example, in describing the operative treatment of gall stones in the common duct, the author simply mentions the various procedures without giving indications for or against the different methods. He does not tell us how to suture the duct after removal of the stone, nor whether to guard the wound in the duodenum with gauze packing after incision. In speaking of the treatment of chronic gonorrhea with involvement of the seminal vesicles, he advises the plan of milking out the discharge by rectal palpation, but omits to say how long or how often to apply the pressure. Abbe's catgut rings for intestinal anastomosis are alluded to, but there is no hint to guide one in making them. In the treatment of flat-foot he says, "much comfort is often given by placing in the shoe a metal plate to restore the arch of the foot," and the reader must look elsewhere for further information, if he cares to employ this device. Ten lines are deemed sufficient to describe the operation of vaginal hysterectomy. After one page is devoted to the operation for hemorrhoids by clamp and cautery, one short paragraph only gives the Alexander operation with the omission of practical points as to suturing the mucous membrane. In other words, the author slights certain few sections and deals fully with the remainder. The whole subject of fractures is admirably handled in every particular. The majority of the illustrations are new, many of them being taken from the author's own cases. The fact that the whole work is written by one man of unquestioned authority, and does not represent the teachings of a dozen of varying ability enhances its value. The book is most attractively bound and printed.

P. LeB.

